# Porous carbon derived from metal–organic framework@graphene quantum dots as electrode materials for supercapacitors and lithium-ion batteries†

**DOI:** 10.1039/c9ra01488h

**Published:** 2019-03-26

**Authors:** Hui Yu, Wenjian Zhu, Hu Zhou, Jianfeng Liu, Zhen Yang, Xiaocai Hu, Aihua Yuan

**Affiliations:** School of Environmental and Chemical Engineering, Jiangsu University of Science and Technology Zhenjiang 212003 China aihua.yuan@just.edu.cn; School of Material Science and Engineering, Jiangsu University of Science and Technology Zhenjiang 212003 China; Shanghai Waigaoqiao Shipbuilding Co., Ltd Shanghai 200137 China; Marine Equipment and Technology Institute, Jiangsu University of Science and Technology Zhenjiang 212003 China

## Abstract

The C@GQD composite was prepared by the combination of metal–organic framework (ZIF-8)-derived porous carbon and graphene quantum dots (GQDs) by a simple method. The resulting composite has a high specific surface area of 668 m^2^ g^−1^ and involves numerous micro- and mesopores. As a supercapacitor electrode, the material showed an excellent double-layer capacitance and a high capacity retention of 130 F g^−1^ at 2 A g^−1^. The excellent long-term stability was observed even after ∼10 000 charge–discharge cycles. Moreover, the composite as an anode material for a lithium-ion battery exhibited a good reversible capacity and outstanding cycle stability (493 mA h g^−1^ at 100 mA g^−1^ after 200 cycles). The synergistic effect of a MOF-derived porous carbon and GQDs was responsible for the improvement of electrochemical properties.

## Abstract 2

The C@GQD composite was prepared by the combination of metal–organic framework (ZIF-8)-derived porous carbon and graphene quantum dots (GQDs) by a simple method.

## Graphical abstract

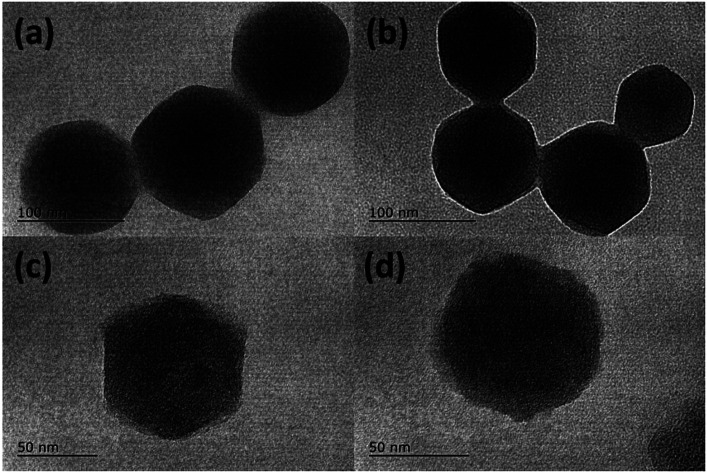

## 1. Introduction

The development and utilization of sustainable energy has gradually become an urgent concern. The energy storage devices of supercapacitors and lithium-ion batteries (LIBs) are of great interest due to their high energy density, excellent rate performance, and outstanding cycle life.^1–4^ Electrode materials are generally considered to be a key factor for the high performance of energy storage devices. Recently, a large number of materials have been explored as electrodes in order to obtain excellent electrochemical performances. Numerous metal oxides,^5–7^ hydroxides,^8–10^ and conductive materials^11–13^ were used for energy applications. Porous carbon not only has the advantage of low cost, high chemical stability and good electrical conductivity, but also has a high specific surface area, adjustable pore structure and large pore volume.^14,15^ Therefore, porous carbon has received considerable interests in the field of energy storage and conversion.^16–18^

Recently, metal–organic frameworks (MOFs) have been widely employed in the preparation of porous carbon because of their large specific surface area, structural diversity, functional pores and high carbon content of organic ligands.^19,20^ However, the electrodes of porous carbon still have limited conductivity and instability because of the decrease in conductivity and large volume change during charge/discharge cycles. As a result, the specific capacity and rate capability are significantly restricted. In order to solve these problems, researchers have devoted to the design and manufacture of nanostructured carbon (carbon nanotubes,^21^ carbon fibers,^22^ graphene,^23^*etc.*), which could be employed to form conductive networks, increasing the conductivity of electrode.

Recently, researchers have developed a new type of carbon material, graphene quantum dots (GQDs), which have extra unique advantages originated from their small size of nanometers,^24^ and then a large amount of GQDs-based composites have been documented.^3^ Mondal *et al.* reported GQDs supercapacitors based on aniline chemical oxidation derivative doped polyaniline complexes.^25^ The GQDP (polyaniline) composites showed a specific capacitance of 1044 F g^−1^ at 1 A g^−1^ after 3000-cycle with capacitance retention rate of 80.1%. Biswal *et al.* presented a simple strategy for the encapsulation and stabilization of GQDs in ZIF-8, resulting in photoluminescence emission even after 3 months.^26^ A work on doping GQD as an anode material has recently been reported. The authors reported a simple strategy to synthesize the N and S co-doped GQDs as negative electrode materials for LIBs. The introduction of (N, S)-doped GQD greatly improved the speed of electron transfer and the capacity of lithium storage. The discharge capacity was 254.2 mA h g^−1^ at 0.1C and was 126.5 mA h g^−1^ at 10C. After the cycle of at least 2000 times at 2C, the retention rate was 96.9%. These results clearly demonstrate that GQD is a promising material for electrochemical applications in comparison to unstable semiconductors. Here, a simple strategy was developed to synthesize porous carbon derived from ZIF-8@GQDs. This is the first case of composites containing MOF-derived carbon and GQDs. The composite could be employed as electrode materials for supercapacitor and LIBs and exhibited excellent electrochemical performances.

## 2. Experimental

### 2.1. Materials

Zinc nitrate hexahydrate (Zn(NO_3_)_2_·6H_2_O, ≥99.0%) and 2-methylimidazole (2-MeIm, ≥99.0%) were available from Aladdin Chemical Co. Methanol was purchased from the Chinese medicine Reagent Co. Imidazole-modified GQDs (1 mg mL^−1^) were obtained from Nanjing XFNANO Co. Ltd, China. All raw materials are commercially available and used without further purification.

### 2.2. Synthesis of ZIF-8 and ZIF-8@GQDs

In a typical synthesis,^27^ 1.312 g (4.4 mmol) of Zn(NO_3_)_2_·6H_2_O was dissolved in 50 mL of methanol to form solution A. 2.898 g (35.3 mmol) of 2-methylimidazole was formed into solution B in 50 mL of methanol. Subsequently, solution A was added to B and stirred at room temperature for 1 h. The white precipitates were collected by centrifuging, washed several times in methanol and dried well under vacuum at 60 °C for 6 h to obtain ZIF-8.

The synthesis of ZIF-8@GQDs precursor was similar to that of ZIF-8 mentioned above except that GQDs solution (4 mL, 1 mg mL^−1^) was added to solution B during the preparation process.

### 2.3. Synthesis of C(ZIF-8) and C(ZIF-8)@GQDs

ZIF-8 derived carbon was prepared by calcining ZIF-8 in a furnace under the flow of N_2_. At first, the calcination temperature was raised to 1000 °C at a heating rate of 10 °C min^−1^, and then maintained at 1000 °C for 3 h. Upon naturally cooling down to room temperature, the carbonized products were collected and ground. The material was finally soaked in 1 mol L^−1^ HCl for 24 h to flush away the remaining Zn or ZnO impurities. The sample was then washed with deionized water to neutrality, centrifuged and dried under vacuum at 80 °C. The resulting black product C(ZIF-8) was finally harvested.

As a control experiment, the synthesis of C(ZIF-8)@GQDs was similar to the process of C(ZIF-8) except the replacement of ZIF-8 by ZIF-8@GQDs.

### 2.4. Characterization

Field emission scanning electron microscope (FE-SEM, ZEISS Merlin Compact) was used to study the morphology and structure of products. The Shimadzu XRD-6000 diffractometer (Cu-K_α_ radiation, 0.15406 nm) was employed to record the phase structure of samples. The nitrogen adsorption–desorption isotherms of materials were recorded at 77 K on a BEL (Japan, Inc.) instrument. Before adsorption measurement, the sample was degassed under vacuum for 12 h at 433 K. Based on the adsorption data by Brunauer–Emmett–Teller (BET) computing specific surface area, and the pore size distribution curve was determined using Barrett–Joyner–Halenda (BJH) method. The element composition and chemical state of the samples were studied by X-ray photoelectron spectroscopy (XPS, Thermo-VG Scientific ESCA-LAB250).

### 2.5. Electrochemical measurements

The working electrode of supercapacitors was prepared by combining the active materials (C(ZIF-8) and C(ZIF-8)@GQDs), conductive agent (Super P) to and polymer binder (polyvinylidene fluoride, PVDF) with mass ratio of 8 : 1 : 1 mixture of *N*-methyl-2-pyrrolidone (NMP) as solvent. The prepared slurry was uniformly applied to the treated nickel foam (1 cm × 1 cm) and dried at 80 °C for 24 h. The resulting work electrode contained approximately 1.5 mg of active material. All electrochemical measurements were carried out at room temperature in a three-electrode system: the treated nickel foam coated with C(ZIF-8)@GQDs was used as the working electrode, Pt foil (1 cm^2^) as the opposite electrode, and the saturated calomel electrode (SCE) as the reference electrode. The electrolyte solution is 6 mol L^−1^ KOH solution. All of these electrochemical measurements were conducted by the Autolab workstation (CHI 660C). The performance of supercapacitors was tested by cyclic voltammetry (CV) and constant current charge–discharge (GCD). Based on the following equation can calculate the specific capacitance (*C*) of the working electrode: *C*_m_ = (*I* × Δ*t*)/(*m* × Δ*V*), in which *C*_m_ [F g^−1^] is the working electrode capacitance calculated according to the active material quality, *I* [A] is the discharge current, *m* [g] is the mass load of active material, Δ*V* [V] is the charge–discharge window.

A coin cell battery (CR2032) was used to test the LIBs performance of materials and the battery was assembled in a glove box filled with argon. Celgard 2600 was used as separator, metal–lithium foil was used as counter electrode, and 1 M LiPF_6_ was used as electrolyte in ethylene carbonate/diethyl carbonate (v : v = 1 : 1). The active materials (C(ZIF-8) and C(ZIF-8)@GQDs), conductive agent (Super P) and polymer binder (polyvinylidene fluoride, PVDF) were combined to form the working electrode with a mass ratio of 8 : 1 : 1 mixture of *N*-methyl-2-pyrrolidone (NMP) as the solvent. The preparation of slurry coated on copper foil substrate, and dried under vacuum at 80 °C for 12 h. A constant current charge/discharge measurement was performed on the LAND CT 2001A cell system. The CHI660D electrochemical measurement cyclic voltammetry (CV) workstation (Chenhua, Shanghai, China) was used at a scan rate of 0.2 mV s^−1^ with a voltage range between 0.01 and 3.0 V.

## 3. Results and discussion

**Fig. 1:**
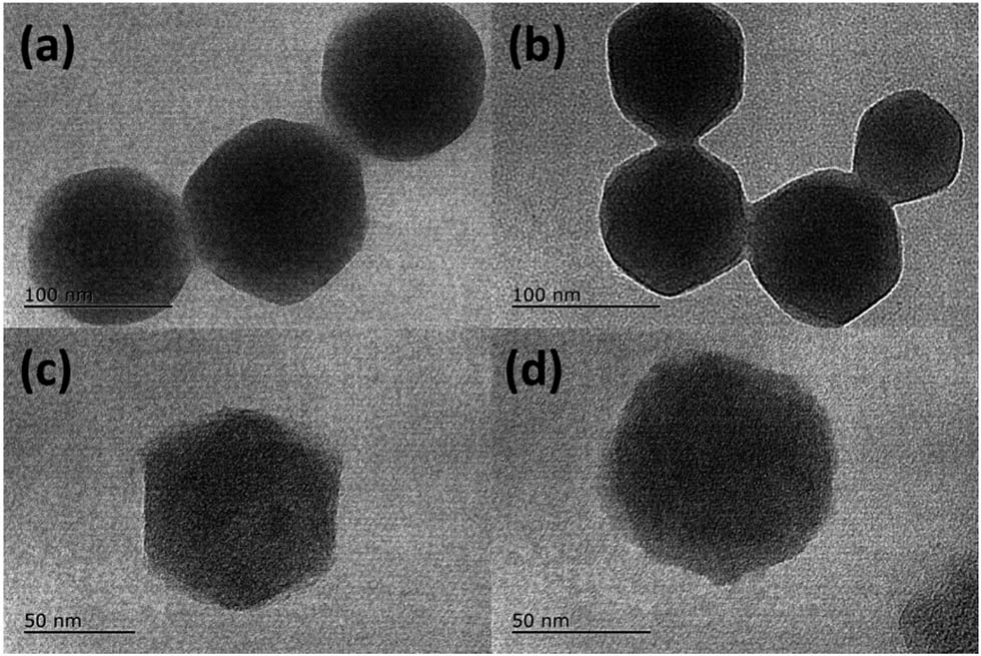
TEM images of (a) ZIF-8, (b) ZIF-8@GQDs, (c) C(ZIF-8), and (d) C(ZIF-8)@GQDs.

SEM images (Fig. S1†) of as-obtained ZIF-8 and ZIF-8@GQDs showed that ZIF-8 exhibited a regular polyhedral morphology with smooth surface. It can be observed from Fig. 1 that ZIF-8@GQDs also presents a hexagonal shape, with particle size of about 100 nm. Since the imidazole-modified GQDs are used in the experiment, the GQDs interact with the metal ions which constitute the framework during the growth process, thereby performing growth self-assembly. From the pore size distribution of ZIF-8 (Fig. S2†), it can also be determined that there are a large number of micropores and mesopores, and GQDs are suitable for entering ZIF-8. We believe that through this *in situ* growth, the GQDs are distributed in the ZIF-8 crystal. This encapsulation process not only passes through simple physical adsorption within the ZIF-8 pore size, but also chemical adsorption with metal ions.^28^ The consistency of size and shape of ZIF-8@GQDs and ZIF-8 indicates that the introduction of GQDs does not significantly change the morphology of pure ZIF-8. According to the elemental mapping result, the distribution of Zn and C elements could be clearly detected (Fig. S3†). After the calcination and etching processes, the surface of the composite became rough and the shape basically remained the same as precursor.

**Fig. 2:**
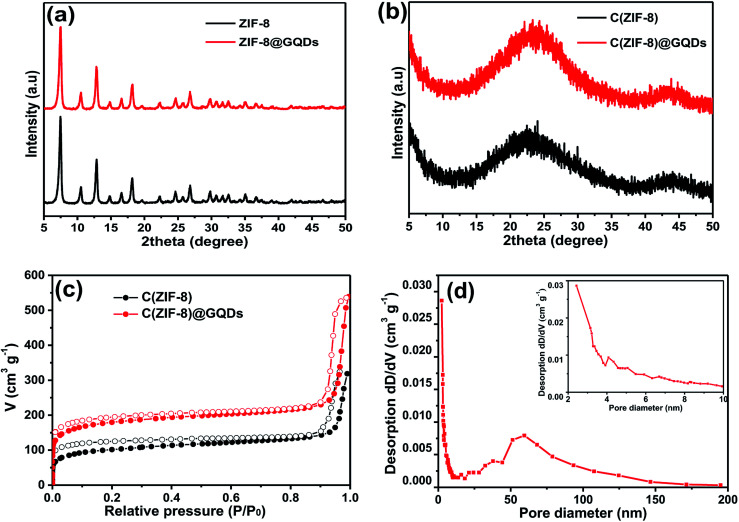
XRD patterns of (a) ZIF-8 and ZIF-8@GQDs, (b) C(ZIF-8) and C(ZIF-8)@GQDs, (c) N_2_ adsorption/desorption isotherms of C(ZIF-8) and C(ZIF-8)@GQDs, (d) pore size distribution of C(ZIF-8)@GQDs.

The phase structure of samples was determined by XRD as shown in Fig. 2a. The ZIF-8 and ZIF-8@GQDs products were highly crystallized with sharp diffraction peaks. The ZIF-8@GQDs composite only showed the characteristic peaks of ZIF-8 and no peaks from GQDs were observed, which is consistent with the previous material. After the calcination and etching, the resulting composite exhibited a typical peak assigned to amorphous carbon (Fig. 2b). This result indicated that the amount of GQD was very low and the peaks were too weak to be detected. As is known to all, the specific surface area is a key factor for electrode materials and faradaic redox reactions. The nitrogen adsorption–desorption measurements were shown in Fig. 2c, which exhibited typical type-I curve with a sharp increase at low pressure (*P*/*P*_0_), indicating the predominant microporous characteristics. The measured BET value of C(ZIF-8)@GQDs was 668 m^2^ g^−1^. Similarly, the isotherm of C(ZIF-8) was type-I with BET value of 378 m^2^ g^−1^. In this experiment, the precursors were treated with high temperature, which led to the change of pore structure. As the added imidazole-modified GQDs were coordinated with more metal ions, the zinc ions were leached out by calcination and etching, resulting in the formation of pore structure. The test results show that the aperture of C(ZIF-8)@GQDs (0.807 cm^3^ g^−1^) is larger than that of C(ZIF-8) (0.4917 cm^3^ g^−1^). This characteristic is more conducive to nitrogen adsorption so as to increase the specific surface area. The results showed that C(ZIF-8)@GQDs successfully inherited the advantage of MOF precursor such as large surface area (Fig. S2†). The increase in the specific surface area of C(ZIF-8)@GQDs may be due to the formation of some mesopores in the high temperature carbonization process with the addition of GQDs. In the high relative pressure zone (*P*/*P*_0_ > 0.9), the adsorption curves of both samples were increased, and there was a significant hysteresis loop, indicating the presence of mesopores. According to TEM images, it can be inferred that the hysteresis loop originates from the physical stacking gap between carbon particles. From the perspective of pore size distribution (Fig. 2d), the mesopores size was mainly distributed at about 2.5 nm, which was relatively uniform and the pore volume is 0.807 cm^3^ g^−1^. Such structural feature for the composite provides more active sites for electrochemical reactions, shortens the ion transport pathways and increases the contact area of electrolyte.

**Fig. 3:**
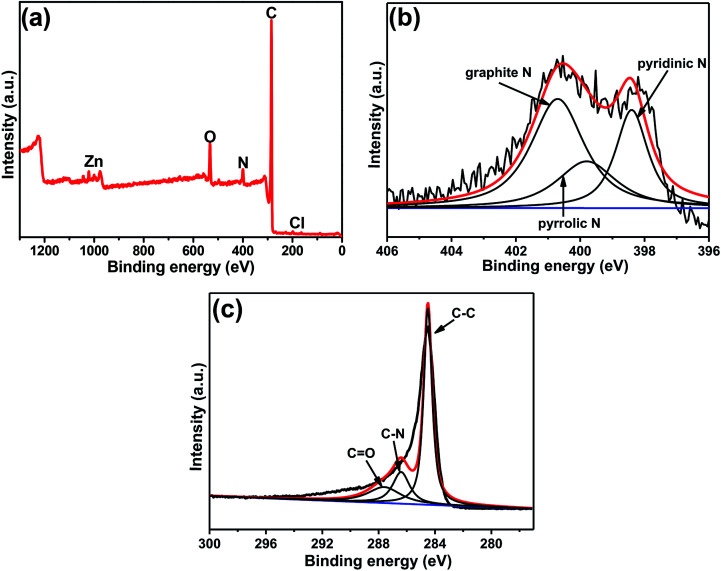
XPS spectra of C(ZIF-8)@GQDs: (a) survey, (b) N 1s, and (c) C 1s.

XPS was used to further detect the elemental composition and species in the composite. As shown in Fig. 3a, it's clear to observe the peaks of C, N and O, with the mass contents of 84.58%, 5.67%, and 8.37%, respectively (Table S1†). It is worth noting that peaks of zinc and chlorine were also observed in the figure, confirming that very small amounts of zinc or zinc compounds were not completely removed by acid etching from the final product. However, no zinc element was found in the carbon obtained from pure ZIF-8, indicating that it may be zinc metal encapsulated by GQDs during the growth process and cannot be completely etched away. The chlorine element is caused by the acid remaining in the washing process. According to the table data (Table S1†), the carbon and nitrogen content of C(ZIF-8)@GQDs is higher than that of C(ZIF-8), which is believed to be caused by the doping of GQDs. The content and form of nitrogen in carbon have an important influence on the electrochemical performance. The XPS spectrum of N 1s can be divided into three different peaks by fitting the curve, corresponding to three forms of nitrogen: pyridinic N, pyrrolic N and graphite N.^29^ It is well known that the content and type of nitrogen affect the electrochemical properties, among which the graphite N has a crucial influence on the electrochemical process than pyridinium N and pyrrole N, because it can improve the conductivity of electrodes.^30^ According to the results shown in Fig. 3b, the C(ZIF-8)@GQDs composite has the highest content of graphite N (49.33%), significantly higher than the graphite N content in C(ZIF-8) (28.83%) (Table S1†). The addition of GQDs greatly increases the degree of graphitization of the material, resulting in a better electrochemical property. The C 1s XP spectrum of C(ZIF-8)@GQDs shows three different types of carbon peaks corresponding to C=C, C–N and C=O, with binding energies of 284.5, 285.6, and 288.0 eV, respectively (Fig. 3c). Compared to the synthesized C(ZIF-8), three peaks were observed for the three different types of carbon (C=C, C-sp^3^ and C–N) present in the C 1s XP spectrum. The binding energy values were 284.0, 284.1 and 286.1 eV, respectively (Fig. S4†). According to the literature reports,^26^ C–C and C=O carbon peaks shown in the C 1s XPS spectrum of GQD were 284.5 and 287.6 eV, respectively. We believe that the extra carbon peak (C=O) in the C(ZIF-8)@GQDs is due to the incorporation of GQDs into the ZIF-8 nanocrystal matrix.

**Fig. 4:**
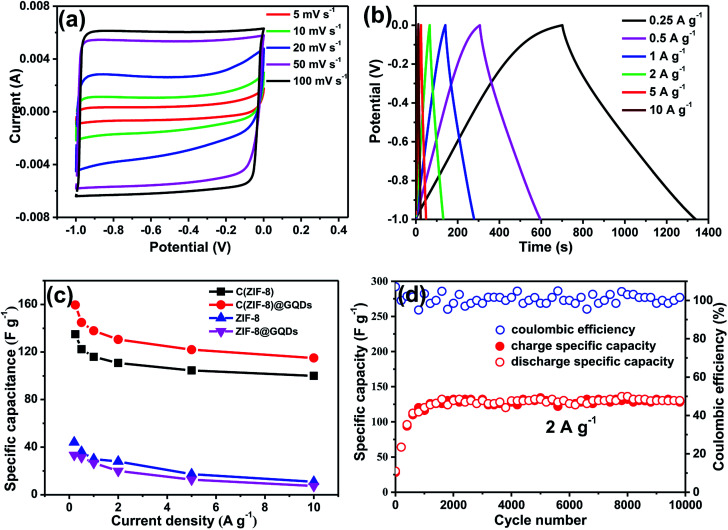
(a) CV curves of C(ZIF-8)@GQDs at different scan rates; (b) GCD curves of C(ZIF-8)@GQDs at different current densities; (c) the specific capacitances of ZIF-8, ZIF-8@GQDs, C(ZIF-8) and C(ZIF-8)@GQDs at different current densities; (d) cycling performance of C(ZIF-8)@GQDs during 9999 cycles at 2 A g^−1^.

Fig. 4a showed the CV curves of C(ZIF-8)@GQDs as supercapacitor electrode at different scan rates. The curves exhibited a rectangular shape, revealing that the electrode has a good double-layer capacitance performance. It can be seen that CV curves still maintained the similar shape when the scan rate was increased from 5 mV s^−1^ to 100 mV s^−1^, indicating a significant electrochemical reversibility. This can be ascribed to the interconnected porous nanostructures and the unique small size effect of GQDs, which allows the electrolyte to enter the active surface of electrode. The C(ZIF-8)@GQDs electrodes measured between −1 and 0 V at different current densities were shown in Fig. 4b. The areas of GCD curves decreased with increasing current densities from 0.25 to 10 A g^−1^. It's found that the GCD curves were triangular, revealing that the composite has an excellent double layer capacitance behaviour. Calculating the GCD curves at different current densities, the specific capacitances of electrodes were provided in Fig. 4c. At the current densities of 0.25, 0.5, 1, 2, 5, and 10 A g^−1^, the specific capacitances were 159.6, 144.8, 137.9, 130.6, 122 and 115 F g^−1^, respectively. The specific capacitance of C(ZIF-8)@GQDs was significantly better than the other three materials (Fig. S5 and S6†). The specific capacity of C(ZIF-8)@GQDs was slightly lower than that of MOF-based porous carbon materials with the same current density reported in the literature.^31–33^ Even at the high current density, the specific capacitance retention of C(ZIF-8)@GQDs at the rate of 10 A g^−1^ was still 72.1%, much higher than those observed in other carbon materials.^34,35^Fig. 4d showed a long cycle test of C(ZIF-8)@GQDs. During the first 1500 cycles, the specific capacitance gradually increased, which can be attributed to the fact that the active material did not completely contact with the electrolyte. After 1500 cycles, the discharge capacity was stabilized at 130 F g^−1^. It is worth noting that the C(ZIF-8)@GQDs electrode still showed excellent cycle capacity retention after 9999 cycles with a coulomb efficiency of almost 100%.

**Fig. 5:**
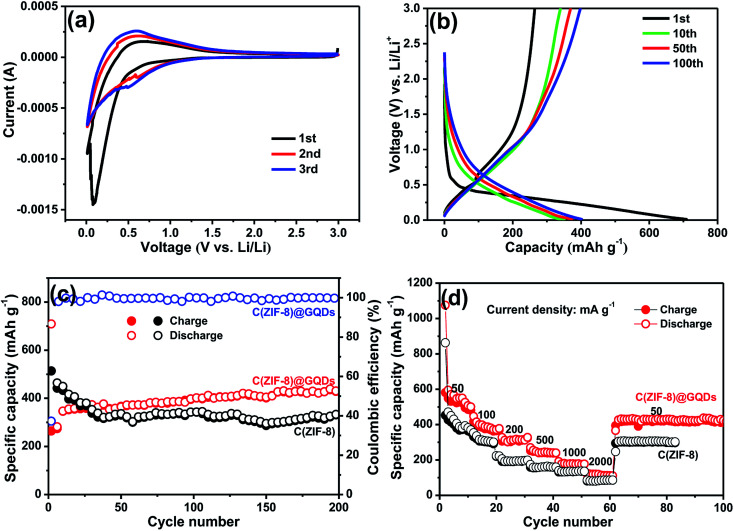
The LIBs performance of C(ZIF-8)@GQDs as anode material: (a) CV curves at a scan rate of 0.2 mV s^−1^ in the voltage range of 0.01–3.0 V, (b) the galvanostatic charge–discharge profiles at 100 mA g^−1^, (c) the cycling stability at 100 mA g^−1^, and (d) the rate capability at different current densities from 50 to 2000 mA g^−1^.

The C(ZIF-8)@GQDs composite was used as the anode material of LIBs to further study the electrochemical properties. The sample was tested at a scan rate of 0.2 mV s^−1^ between 0.1 and 3.0 V at room temperature. The first CV curve (Fig. 5a) was different from the subsequent cycle, especially for the discharge curve. The peak intensity of the first lap was much stronger than the subsequent one, suggesting that some irreversible reactions occurred and the SEI film was formed.^36^ Based on the analysis of the 2nd and 3rd cycles, both CV curves were almost overlapped, thereby ensuring the good cyclic stability and capacity reversibility of electrode. The discharge/charge curves of C(ZIF-8)@GQDs at 100 mA g^−1^ (0.1–3.0 V) were shown in Fig. 5b. The discharge capacity was up to 708 mA h g^−1^ in the first cycle, with a reversible specific capacity of 264 mA h g^−1^ and the initial coulombic efficiency of about 37.29%. The initial irreversible loss of capacity resulted in low Coulomb efficiency indicating the decomposition of SEI film and electrolyte. The results are also consistent with above CV data, where the cathode peak appeared in the first scan but did not exist in subsequent ones. The cycle stability of C(ZIF-8)@GQDs was evaluated at 100 mA g^−1^, as shown in Fig. 5c. The composite has a better cycle performance than C(ZIF-8). The first 50 laps were unstable and slowly rising, probably due to the fact that the electrolyte not completely saturating the pads. After 200 cycles, the electrode holds a discharge capacity of 493 mA h g^−1^. When pure C(ZIF-8) was used as anode material, the specific capacity can only be maintained at 329 mA h g^−1^. It is proved by the comparison that the coating of GQDs plays an important role for the improvement electrochemical performance. Fig. 5d showed the rate capability of C(ZIF-8)@GQDs at various current densities. When the current density changed from 50 to 100, 200, 500, 1000 and 2000 mA g^−1^, the corresponding discharge capacities were about 546.0, 388.0, 313.7, 246.6, 178.7 and 115.5 mA h g^−1^, respectively. The specific capacities of C(ZIF-8) under the same conditions were 406.9, 315.8, 194.5, 164.2, 134.7 and 85.6 mA h g^−1^. Obviously, C(ZIF-8)@GQDs electrode is significantly superior to C(ZIF-8) in capacity. When the current density returned to 50 mA g^−1^, the discharge capacity reached about 425 mA h g^−1^, demonstrating the composite has an excellent rate capability.

## 4. Conclusions

In general, we reported an effective method to combine MOF and GQDs were successfully by *in situ* self-assembly method, and a new type of porous carbon material was finally prepared by high-temperature calcination and etching. These operations result in changes in pore structure, specific surface area, and element content. This feature can improve the migration rate of electrolyte ions in supercapacitors and accelerate the embedding and de-embedding of lithium ions in lithium-ion batteries. As a supercapacitor electrode, the material delivered excellent rate capability and long cycle stability. The discharge capacity was still stable at 130 F g^−1^ at 2 A g^−1^ after 9999 cycles. Furthermore, the composite can also be used as anode material of LIBs, showing high reversible capacity and good cycle stability. Thus, the combination of MOF-derived porous carbon and GQDs provides a new method for the design and synthesis of electrode materials for energy conversion/storage devices.

## Conflicts of interest

There are no conflicts to declare.

## Supplementary Material

RA-009-C9RA01488H-s001
