# Supplementary material for: Porous carbon derived from metal–organic framework@graphene quantum dots as electrode materials for supercapacitors and lithium-ion batteries
Source: RSC Adv. 2019 Mar 26;9(17):9577–83. doi: 10.1039/c9ra01488h (PMC9062154; doi:10.1039/c9ra01488h)
Supplement: RA-009-C9RA01488H-s001 [file RA-009-C9RA01488H-s001.pdf]

Electronic Supplementary Material (ESI) for RSC Advances.
This journal is © The Royal Society of Chemistry 2019

Supporting information for

Porous carbon derived from metal-organic framework@graphene quantum dots as electrode materials for supercapacitors and lithium-ion batteries

Hui Yu,[a] Wenjian Zhu,[a] Hu Zhou,[b] Jianfeng Liu,[*,c] Zhen Yang,[c] Xiaocai Hu,[c] Aihua Yuan,[*,a,d]

[a] *School of Environmental and Chemical Engineering, Jiangsu University of Science and Technology, Zhenjiang 212003, China. E-mail: aihua.yuan@just.edu.cn.*

[b] *School of Material Science and Engineering, Jiangsu University of Science and Technology, Zhenjiang 212003, China.*

[c] *Shanghai Waigaoqiao Shipbuilding Co., LTD, Shanghai 200137, China.*

[d] *Marine Equipment and Technology Institute, Jiangsu University of Science and Technology, Zhenjiang 212003, China.*

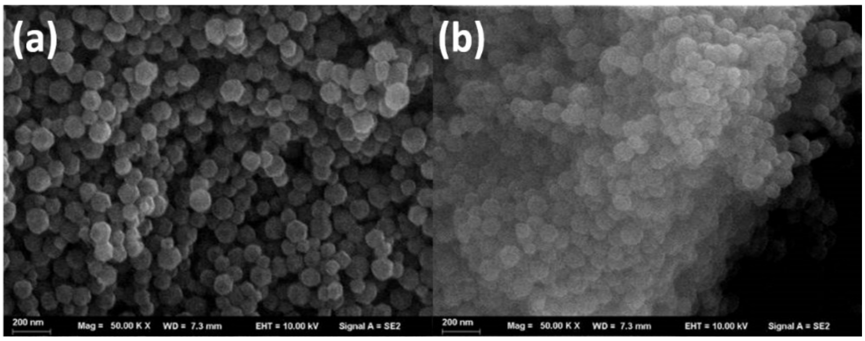

Fig. S1 SEM images of (a) ZIF-8 and (b) ZIF-8@GQDs.

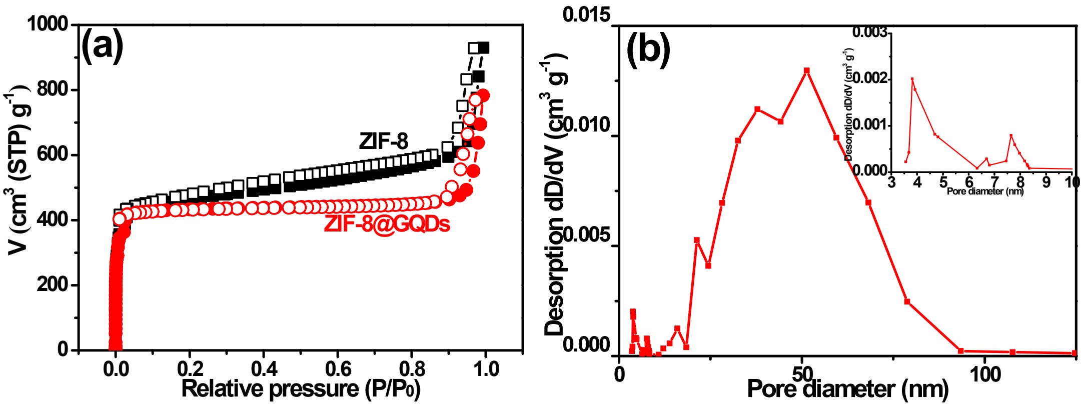

Fig. S2 (a) The N_2 adsorption-desorption isotherm of ZIF-8 and ZIF-8@GQDs; (b) pore size distribution of ZIF-8

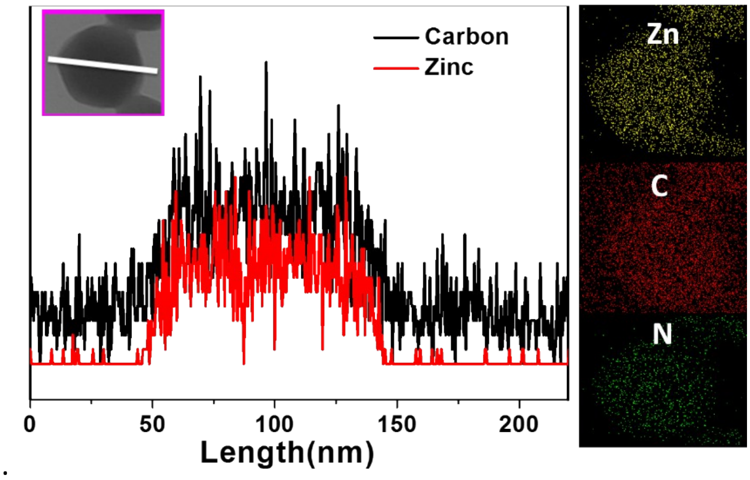

Fig. S3 Elemental line profiles and elemental mapping of ZIF-8@GQDs.

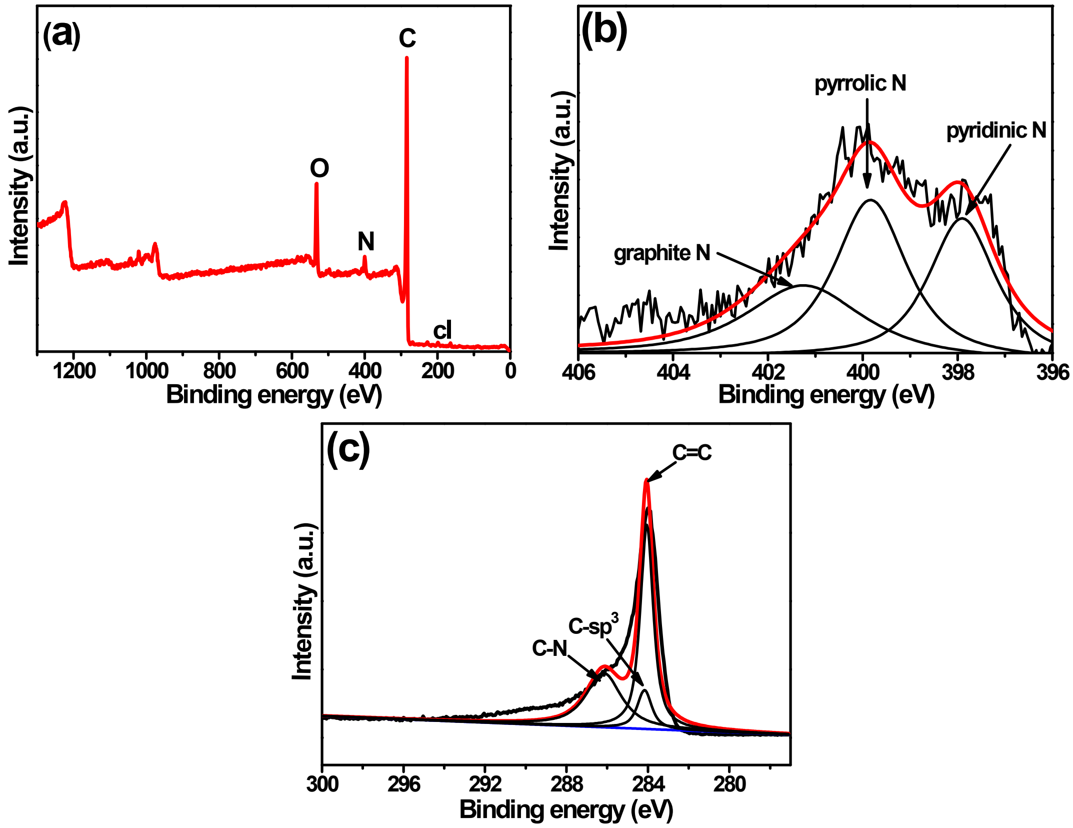

Fig. S4 XPS spectra of C(ZIF-8);(a)survey; (b) N 1s; (c) C 1s.

Table 1 The composition of the obtained porous carbon samples.

C(ZIF-8)@GQDs	XPS (%)					N 1s (%)		
	C	N	O	Zn	cl	graphite N	pyrrolic N	pyridinic N
	84.58	5.67	8.37	0.53	0.85	49.33	23.64	27.03
C(ZIF-8)	82.31	5.39	11.78	0	0.53	28.83	38.83	32.33

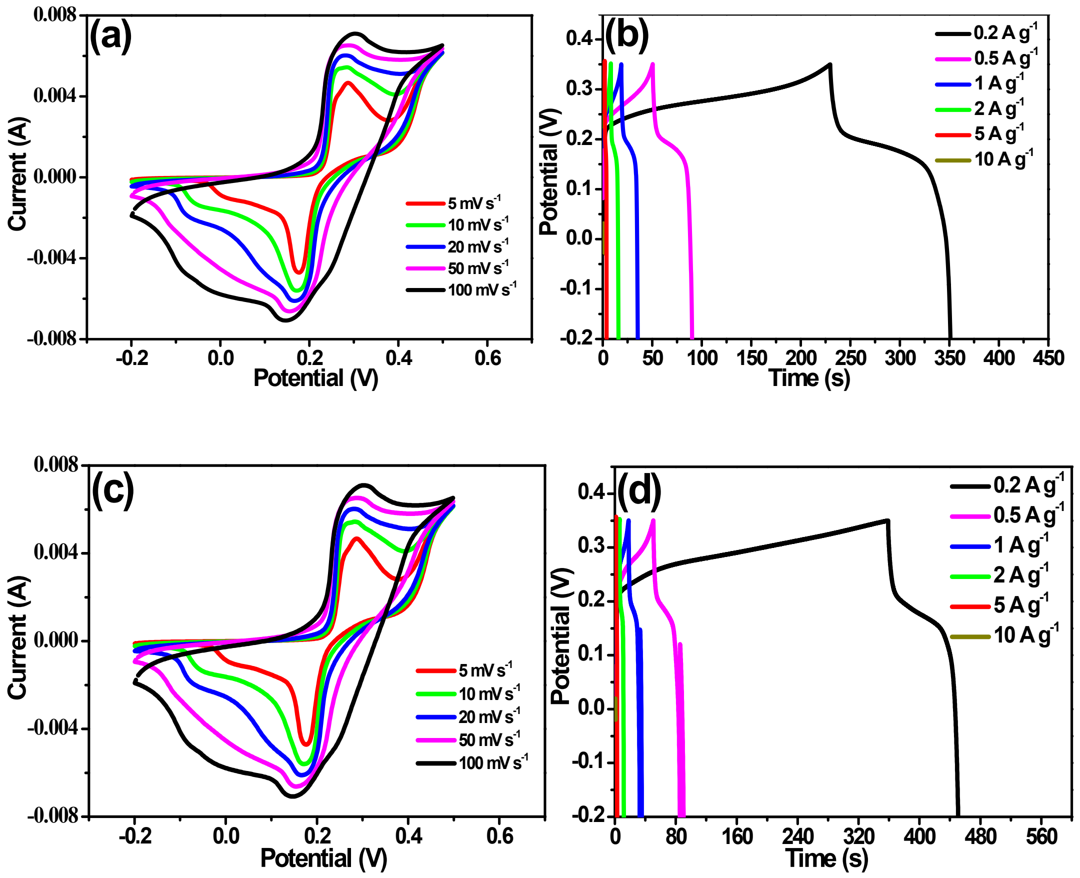

Fig. S5 (a) CV curves of ZIF-8 at different scan rates; (b) GCD curves of ZIF-8 at different current (c) CV curves of ZIF-8@GQDs at different scan rates; (d) GCD curves of ZIF-8@GQDs at different current densities.

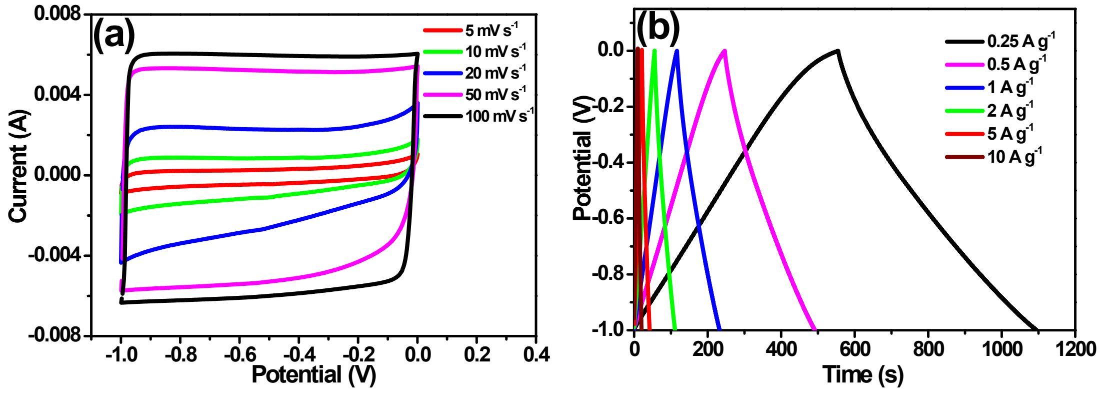

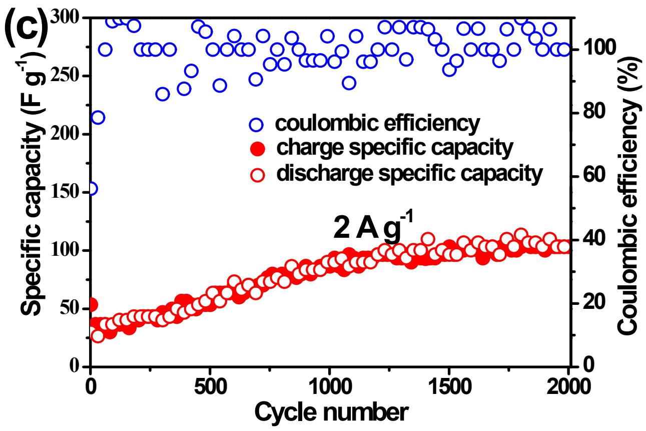

Fig. S6 (a) CV curves of C(ZIF-8) at different scan rates; (b) GCD curves of C(ZIF-8) at different current densities; (c) cycling performance of C(ZIF-8) at 2 A g^{-1}.
